# Modelling of SEPIC, Ćuk and Zeta Converters in Discontinuous Conduction Mode and Performance Evaluation

**DOI:** 10.3390/s21227434

**Published:** 2021-11-09

**Authors:** Emerson Madrid, Duberney Murillo-Yarce, Carlos Restrepo, Javier Muñoz, Roberto Giral

**Affiliations:** 1 Master’s Program in Energy Conversion, Faculty of Engineering, Universidad de Talca, Curicó 3340000, Chile; emerson.madrid@utalca.cl; 2 Engineering Systems Doctoral Program, Faculty of Engineering, Universidad de Talca, Curicó 3340000, Chile; 3 Faculty of Engineering, Universidad de Talca, Curicó 3340000, Chile; crestrepo@utalca.cl (C.R.); jamunoz@utalca.cl (J.M.); 4 Departament d’Enginyeria Electrònica, Elèctrica i Automàtica, Escola Tècnica Superior d’Enginyeria, Universitat Rovira i Virgili, 43003 Tarragona, Spain; roberto.giral@urv.cat

**Keywords:** discontinuous conduction mode, full-order dynamic models, high-order switched converters

## Abstract

High-order switched DC-DC converters, such as SEPIC, Ćuk and Zeta, are classic energy processing elements, which can be used in a wide variety of applications due to their capacity to step-up and/or step-down voltage characteristic. In this paper, a novel methodology for analyzing the previous converters operating in discontinuous conduction mode (DCM) is applied to obtain full-order dynamic models. The analysis is based on the fact that inductor currents have three differentiated operating sub-intervals characterized by a third one in which both currents become equal, which implies that the current flowing through the diode is zero (DCM). Under a small voltage ripple hypothesis, the currents of all three converters have similar current piecewise linear shapes that allow us to use a graphical method based on the triangular shape of the diode current to obtain the respective non-linear average models. The models’ linearization around their steady-state operating points yields full-order small-signal models that reproduce accurately the dynamic behavior of the corresponding switched model. The proposed methodology is applicable to the proposed converters and has also been extended to more complex topologies with magnetic coupling between inductors and/or an RC damping network in parallel with the intermediate capacitor. Several tests were carried out using simulation, hardware-in-the-loop, and using an experimental prototype. All the results validate the theoretical models.

## 1. Introduction

Discontinuous conduction mode (DCM) appears in current unidirectional DC-DC elementary switching converters such as buck, boost or buck-boost, whose switch consists of a transistor (usually a MOSFET or an IGBT) and diode, when the inductor current becomes zero in the diode conduction subinterval. The diode turns OFF and a third subinterval appears in which both transistor and diode are OFF [1,2]. In these converters a continuous conduction mode (CCM) is easily achieved by using synchronous switching, that is, replacing the diode with a transistor and applying complementary switching signals to the resulting current-bidirectional half-bridge structure [1,2].

However, the unidirectional switch is easier to implement and sometimes DCM operation is preferred because it reduces problems related to the diode reverse recovery current [3]. Further, DCM operation in boost and buck-boost converters has been proposed to eliminate the right half-plane zero that can limit the voltage control loop bandwidth [3]. In addition, paralleling DCM converters (with or without interleaving) is achieved naturally without a dedicated control strategy [4].

In single phase AC-DC power factor correction (PFC) applications based on the boost converter, the inductor current becomes discontinuous in the vicinity of the zero-crossings of the line voltage. In fact, some PFC circuits are intentionally designed to always operate in DCM to simplify their control law [3,5,6,7,8] or to reduce the switching loss and the power consumption [9]. The loss free resistor characteristics of the buck-boost converter operating in DCM exhibits a resistive input impedance naturally providing PFC, however because of the discontinuous nature of the buck-boost input current it is sometimes replaced by higher order structures with a series-inductor at its input, such as Single-Ended Primary-Inductor converter (SEPIC) and Ćuk converters [10,11].

Zeta converter is another high order step-up/step-down topology with a switch in series at its input, reason why it has received less attention [12]. The switching cell of the SEPIC, Ćuk and Zeta converters can be easily derived from the buck-boost cell. It has two inductors and an additional intermediate capacitor in addition to the transistor and diode. Since the intermediate and the additional output filter capacitors are usually designed to operate with small voltage ripples, a high frequency transformer can also be added to the basic topologies to provide electrical isolation between the power grid and the load. Because of their high order, all these step-up/step-down converters (SEPIC, Ćuk and Zeta) can exhibit several discontinuous conduction modes [13]. The most usual DCM operation occurs when the diode turns OFF without the MOSFET still turning ON. At this instant, the sum of the two inductor currents which corresponds to the diode current becomes zero [14].

In the literature there are many applications of high order converters such as the basic and modified versions from SEPIC, Ćuk and Zeta topologies operating in DCM. The applications include renewable energies, supplying LED loads, electronic systems, motor drives and electrical vehicles, among others. In the case of the SEPIC converter, it has been the most used in applications like three phase rectifier [11], low power and high power factor correctors [10,15,16], interleaved with coupled inductors [4], PFC with higher output voltage [17] and DC-DC conversion with high voltage gain [18]. In the case of the Ćuk converter the most common applications are bridgeless PFCs [15,19,20] and motor drives [21]. A particular high-order topology combines Ćuk and SEPIC converters to be used in a switched reluctance motor drive [22]. Isolated SEPIC and Ćuk converters are usually in two-stages buck-boost inverters [23]. Finally, in the case of the Zeta converter, there are applications reported in topics such as renewable sources [24], power supplies [25] and electric vehicle battery chargers [26].

The dynamic analysis of the converters is an important topic to design controllers that guarantee the correct operation of the systems. Therefore, it is important to have dynamic models that accurately reproduce the behavior of the switched converter. Modelling methodologies in DCM have been addressed by following analytical or equivalent circuit approaches. On the one hand, the main analytical approach, proposed by Ćuk and Middlebrook, is based on a unified representation of state spaces [2]. This analytical methodology has been used also in high-order converters [3,14,27,28]. On the other hand, the equivalent circuit methodology is an approach which is known in literature as generalized switch averaging technique, first proposed by Vorperian [6]. This methodology applies directly to converters in DCM having a canonical three-terminal switch-cell constituted by the active and passive switch pair. Connecting the canonical cell to a single inductor conforms the core of the well-known basic converters buck, boost and buck-boost. The canonical cell also appears in the Ćuk converter but not in the SEPIC and Zeta converters. The direct application of Vorperian’s analysis methodology requires the converter to have a common-common configuration for the DCM switch. An example of this methodology is the model of the modified Zeta converter presented in [29].

Analytical and equivalent circuit approaches can be used to obtain reduced or full-order models as is proposed in a complete analysis with comparisons of both approaches in [3]. The main feature of reduced-order models is that they are only capable of accurately predicting the converter dynamics at low frequencies. For instance, the model presented in [27] can accurately predict small-signal responses up to one-tenth of the switching frequency.

**Figure 1:**
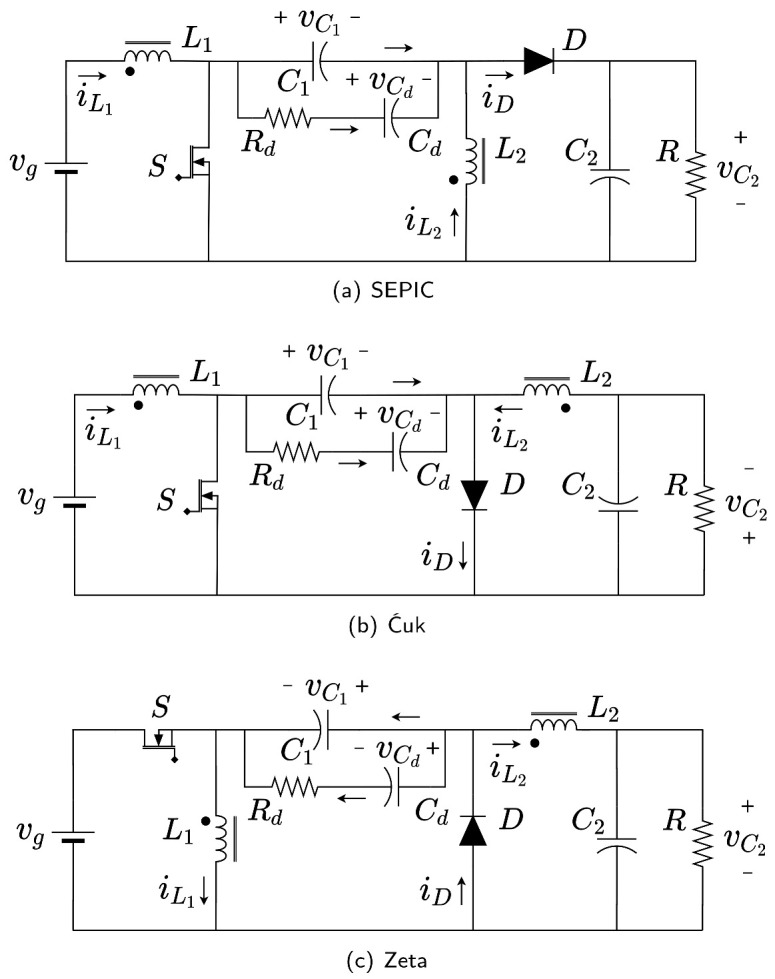
High-order converters: (**a**) SEPIC, (**b**) Ćuk, (**c**) Zeta.

After an intense search of high-order converters in the literature, it is concluded that the scientific community is more focused on the application than on the modelling of high order converter operating in DCM. The motivation of this research emerges from the fact that the methodology proposed in [28] to model the DCM of the Ćuk converter does not work properly in the case of the SEPIC converter in DCM. There were problems to identify a key current component denoted as $i_{x}$ in the differential equation of the intermediate capacitor voltage. In this work, a novel full-order method based on including explicitly the diode average current in the system equations is proposed. The diode average current is obtained for the SEPIC converter by means of a graphical procedure adapted from the procedure followed for the Ćuk converter in [28] and for the interleaved converters in [30]. The use of the diode average current allows us to extend easily the model developed for the SEPIC converter to the Ćuk and Zeta converters. In fact, it provides better results for the Ćuk converter than using the model in [28]. In practice, it is a common procedure to magnetically couple the inductors to reduce from two to one the magnetic core to implement both inductors [31]. Another typical procedure is adding a damping network to improve the transient converter behavior. The damping network is a simple way to minimize the settling time and suppress current and voltage spikes preserving the semiconductor devices. It is also well known that the addition of a damping network in parallel to some capacitors considerably improves the converter’s dynamics (see Chapter 16 in [1]). Another example of the improvement of a converter’s dynamics can be found in [32], where the use of magnetic coupling and a damping network moves the right half plane zeroes to the left half plane in a similar high-order switched converter operating in CCM. The prize to pay for the mentioned improvements is a slightly reduced conversion efficiency due to the added resistive losses in the damping network. In all the cases, the model proposed in this work can also be easily adapted to versions of the SEPIC, Ćuk and Zeta converters depicted in Figure 1 in which the inductors are magnetically coupled and/or an RC damping network is added in parallel to the intermediate capacitor. The main benefits of the proposed methodology are listed below:

- Because it is based in simple graphical representations of inductor’s and diode current waveforms it is easy to understand and apply;
- Provides a full-order model that can be particularized to any of the three high-order step up/down switching converters with or without positive/negative magnetic coupling between inductors and damping networks in the intermediate capacitor;
- The three converters and its variants can be analyzed in the classical forms depicted in Figure 1, where the MOSFET and diode do not share any common node. To apply Vorperian’s method, which also provides full-order models, the structures of two of the converters must be modified to a “common–common” configuration of the DCM switch.

This paper is a contribution to full-order modeling of the SEPIC, Zeta, and Ćuk DCM converters, and it includes simulation, hardware-in-the-loop, and experiments in power circuits to validate the obtained models. Most works in the literature perform only theoretical analysis and simulations. The remainder of the article is organized as follows: Section 2 proposes the modelling procedure, first in a detailed way for the SEPIC converter, and later its generalization to Ćuk and Zeta converters. The model performance in terms of frequency and transient responses are verified in Section 3 by comparing the theoretical predictions with ideal simulations of the switched converters using PSIM software. In addition, hardware-in-the-loop tests and experimental results are presented. Finally, the conclusions and proposal for future works are discussed in Section 4.

## 2. Modeling in Discontinuous Mode of SEPIC, Ćuk and Zeta Converters

The proposed modelling procedure will be applied first in a detailed manner to the SEPIC converter. Afterwards, the method is generalized to the above mentioned high-order converters in DCM: Ćuk and Zeta. The non-linear model provided initially by the proposed method, that will be linearized around the steady-state equilibrium point, has considered the following assumptions:

- Ideal no-losses components, without parasitics;
- Constant switching frequency $f_{s}$ and period *T*;
- Capacitors large enough so that their average voltages can be considered approximately constant through a switching cycle and small voltage ripple amplitudes.

### 2.1. Analysis and Modeling of SEPIC Converter in DCM

**Figure 2:**
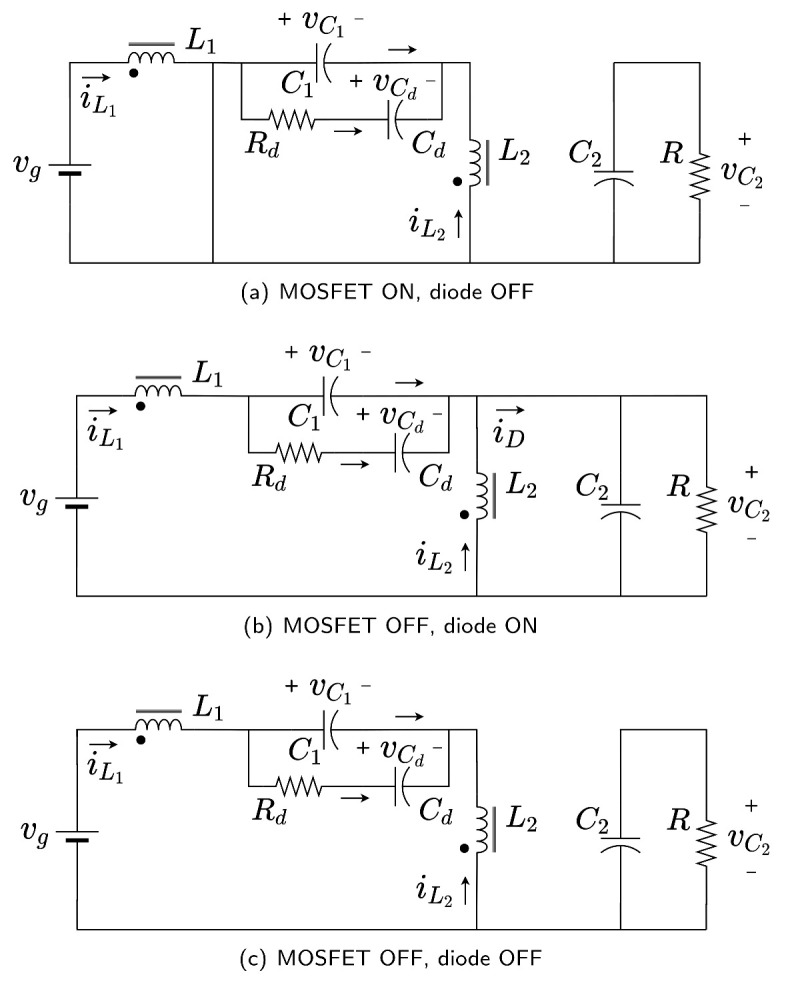
SEPIC converter circuits on DCM operation: (**a**) MOSFET ON, diode OFF, (**b**) MOSFET OFF, diode ON, (**c**) MOSFET OFF, diode OFF.

The proposed methodology starts with the obtention of three ideal subcircuits assuming DCM depicted in Figure 2 from which, by following Kirchhoff’s voltage laws, the inductor current slopes at each subinterval are obtained (1) and ( 2). Subcircuits in Figure 2 are defined by the conduction states of the converter semiconductors (MOSFET and diode) along a switching cycle of constant duration *T*. At the first subinterval, whose duration is $d_{1}T$, the MOSFET is ON and the diode is OFF (Figure 2a). The duration of the second subinterval is $d_{2}T$. In this subinterval (Figure 2b) the MOSFET is turned OFF and the energy stored at the inductors produce the diode activation. Finally, with the MOSFET still OFF, a third subinterval (DCM) appears when both inductor currents combine so that the diode current is zero and the diode turns OFF (Figure 2c). The duration of the third subinterval in which both switches’ semiconductors are OFF is $\left(1-(d_{1}+d_{2})\right)T$. The state variables of the model are average inductor currents and capacitors voltages ${\bar{i}}_{L_{1}}$, ${\bar{i}}_{L_{2}}$, ${\bar{v}}_{C_{1}}$, ${\bar{v}}_{C_{2}}$, and ${\bar{v}}_{C_{d}}$. Magnetic coupling *M* between inductors $L_{1}$ and $L_{2}$ has been considered. A damping ($R_{d}C_{d}$) network connected in parallel to the intermediate capacitor has also been taken into account as shown in Figure 2.

(1) $$\frac{d}{dt}i_{L_{1}}=\left\{\begin{array}{cc} m_{11}, & 0⩽t<d_{1}T\\ m_{21}, & d_{1}T⩽t<\left(d_{1}+d_{2}\right)T\\ m_{3}, & (d_{1}+d_{2})T⩽t<T \end{array}\right.$$

(2) $$\frac{d}{dt}i_{L_{2}}=\left\{\begin{array}{cc} -m_{12}, & 0⩽t<d_{1}T\\ -m_{22}, & d_{1}T⩽t<\left(d_{1}+d_{2}\right)T\\ -m_{3}, & (d_{1}+d_{2})T⩽t<T. \end{array}\right.$$

**Figure 3:**
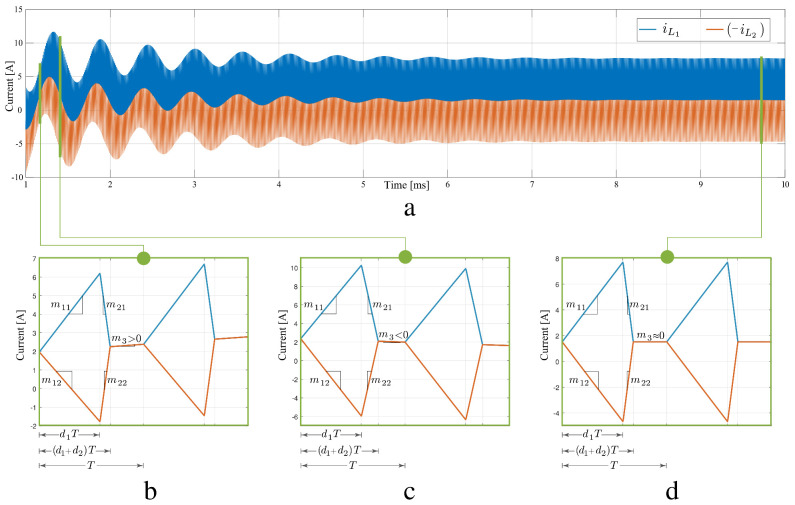
Responses in inductor currents: (**a**) dynamic response for inductor currents, (**b**) current transient with positive slope, (**c**) current transient with negative slope, (**d**) currents in steady state.

Figure 3a shows a simulation of the two inductor current waveforms in which the converter starts up from zero initial conditions under a constant duty cycle control signal (open loop). Three main behaviours can be observed in the enlarged sections in the bottom plots: in Figure 3b the currents increase in a switching cycle, they decrease in the Figure 3c, finally Figure 3d corresponds to a steady-state situation. The derivatives of the averaged inductor currents are obtained as a linear combination of the three current slopes weighted by the relative duration of their associate subintervals, in accordance with the piece-wise waveforms depicted in Figure 3b–d. Current slopes have been denoted as $m_{ij}$, where subindex *i* corresponds to the subinterval number and subindex *j* to the inductor. A particular case are slopes in the third subintervals, since they are equal for both currents they have been renamed with just one subindex as $m_{31}=m_{32}=m_{3}$.

The current slopes, obtained from Figure 2 using Kirchhoff voltage law, have been summarized in Table 1. As expected, the slopes depend on the average voltages and the parameters of the magnetic elements ($L_{1}$, $L_{2}$ and *M*) compacted by using additional parameters ΔL and $L_{S}$, the determinant of the magnetic parameter matrix and the equivalent inductance in the DCM subinterval, respectively.

(3) $$\begin{array}{cc} \; & \frac{d}{dt}{\bar{i}}_{L_{1}}=m_{11}d_{1}+m_{21}d_{2}+m_{3}\left(1-d_{1}-d_{2}\right)\\ \; & \frac{d}{dt}{\bar{i}}_{L_{2}}=-\left(m_{12}d_{1}+m_{22}d_{2}+m_{3}\left(1-d_{1}-d_{2}\right)\right)\\ \; & \frac{d}{dt}{\bar{v}}_{C_{1}}=\frac{1}{C_{1}}\left({\bar{i}}_{D}-{\bar{i}}_{L_{2}}-\frac{1}{R_{d}}\left({\bar{v}}_{C_{1}}-{\bar{v}}_{C_{d}}\right)\right)\\ \; & \frac{d}{dt}{\bar{v}}_{C_{2}}=\frac{1}{C_{2}}\left({\bar{i}}_{D}-\frac{{\bar{v}}_{C_{2}}}{R}\right)\\ \; & \frac{d}{dt}{\bar{v}}_{C_{d}}=\frac{1}{R_{d}C_{d}}\left({\bar{v}}_{C_{1}}-{\bar{v}}_{C_{d}}\right). \end{array}$$

The inductor current average state equations obtained as described previously are the first two of the non-linear model in (3). The remaining equations in the model are the average capacitor voltages that are derived from Kirchhoff current laws. In the equations corresponding to intermediate capacitor $C_{1}$ and output capacitor $C_{2}$, the proposed model introduces the diode average current (${\bar{i}}_{D}$) and the relative duration of the second subinterval ($d_{2}$) as auxiliary key variables that will be determined graphically in the next subsection. As will be seen, these two variables contribute significantly to the non-linearity of the model, that has as inputs the relative duration of the first subinterval ($d_{1}$) and the assumed constant input voltage value (${\bar{v}}_{g}=V_{g}$). Note that $d_{1}$ represents the nominal duty cycle of the converter.

### 2.2. Nonlinear Model of Average Values Based on a Graphical Method for the SEPIC Converter in DCM

The full-order non-linear model of average values of the SEPIC converter shown in (3) allows us to analyze the converter behavior in the operation interval. In general, the inductors currents have a damped oscillatory behavior (see Figure 3a) where, as mentioned previously, it is possible to identify three operating conditions as a function of the average slope: current transient with positive slope (Figure 3b), current transient with negative slope (Figure 3c) and inductor current in steady state (Figure 3d) with zero slope. In Figure 3b it is observed how the average values of the currents increase. This behavior is directly associated with the positive sign of the $m_{3}$ slope. Likewise, in Figure 3c the current average values decrease ($m_{3}<0$) while in Figure 3d the average currents reach a steady state value ($m_{3}\approx 0$). It is important to note in Figure 3 that $i_{L_{2}}$ has been intentionally plotted with a negative sign to show that in the third sub-interval, slopes of $i_{L_{1}}$ and $-i_{L_{2}}$ have equal magnitude ($m_{3}$) as seen in (1) and (2).

In steady state, inductor currents exhibit slopes of opposite signs in the first and second subintervals, and zero slope in the third subinterval ($m_{3}=0$). The slopes and durations of the first and second subintervals result in a constant average inductor current. Due to the rectilinear behavior of the inductor currents in each subinterval, it is possible to obtain their average values from the areas of the triangles between the curves and the horizontal axis. In (4), a general expression of the average inductor current is shown, where $m_{1j}$ is the slope of the inductor current in the first sub-interval and $I_{3}$ is the constant value of the current in the third sub-interval.

(4) $${\bar{i}}_{L_{j}}=\frac{1}{T}\int_{0}^{T}i_{L_{j}}\;dt=\frac{1}{2}m_{1j}d_{1}T\left(d_{1}+d_{2}\right)\pm I_{3}.$$

**Figure 4:**
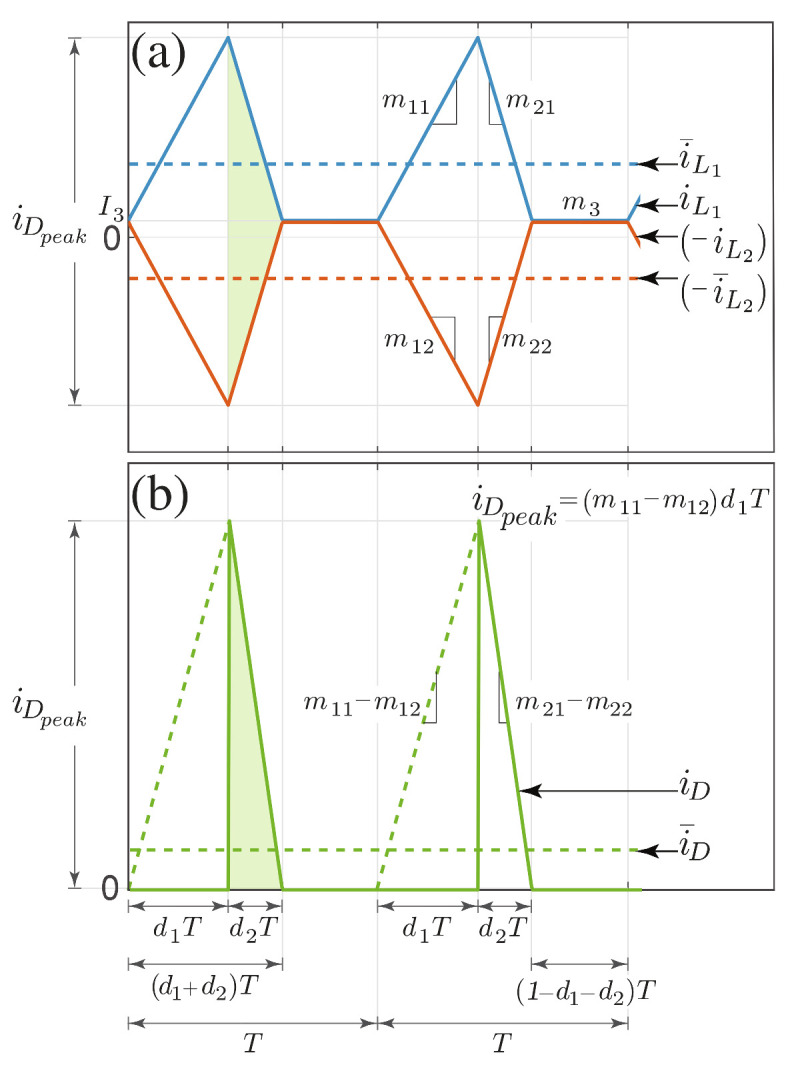
Relation between inductor and diode currents in steady state: (**a**) inductor currents, (**b**) diode current.

Replacing the slope and evaluating the sign of $I_{3}$ in (4), Equations (5) and (6) are obtained. Constant $I_{3}$ is eliminated by adding the previous equations so that the second subinterval duration is obtained (7). On the other hand, (8) allows calculation of the average diode current. This equation is obtained by adding the average inductor currents in the second subinterval. ${\bar{i}}_{D}$ is proportional to the area between the inductor currents as shown in Figure 4a and redrawn as a right triangle in Figure 4b.

(5) $$\begin{array}{cc} {\bar{i}}_{L_{1}} & =\frac{1}{2}m_{11}d_{1}T\left(d_{1}+d_{2}\right)+I_{3} \end{array}$$

(6) $$\begin{array}{cc} {\bar{i}}_{L_{2}} & =\frac{1}{2}\left(-m_{12}\right)d_{1}T\left(d_{1}+d_{2}\right)-I_{3} \end{array}$$

(7) $$\begin{array}{cc} d_{2} & =\frac{2({\bar{i}}_{L_{1}}+{\bar{i}}_{L_{2}})}{\left(m_{11}-m_{12}\right)d_{1}T}-d_{1} \end{array}$$

(8) $$\begin{array}{cc} {\bar{i}}_{D} & =\frac{1}{2}\left(m_{11}-m_{12}\right)d_{1}d_{2}T. \end{array}$$

Since the nonlinear model will be later linearized around the steady-state equilibrium point, it has been assumed that the proposed method, which derives the different average current values from the triangular areas in the steady-state waveforms, will provide simple and precise enough expressions of $d_{2}$ (7) and ${\bar{i}}_{D}$ (8) to complete the model in (3). Both expressions will introduce additional nonlinearities to the dynamical model of the SEPIC converter.

### 2.3. Steady State Operating Point of the SEPIC Converter in DCM

The steady state operating point (9) has been obtained by replacing (7) and (8) in (3) and equating the derivatives to zero.

(9) $$\begin{array}{cc} {\bar{i}}_{L_{1}} & =\frac{{\bar{v}}_{g}T}{2L_{E}}d_{1}^{2}\\ {\bar{i}}_{L_{2}} & ={\bar{i}}_{D}=\frac{{\bar{v}}_{g}}{R}\left(\frac{d_{1}}{d_{2}}\right)\\ {\bar{v}}_{C_{1}} & ={\bar{v}}_{g}\\ {\bar{v}}_{C_{2}} & =R{\bar{i}}_{L_{2}}={\bar{v}}_{g}\left(\frac{d_{1}}{d_{2}}\right)\\ {\bar{v}}_{C_{d}} & ={\bar{v}}_{C_{1}}={\bar{v}}_{g}, \end{array}$$

where

(10) $$\begin{array}{cc} L_{E} & =\frac{\Delta L}{L_{s}}\\ d_{2} & =\sqrt{\frac{2L_{E}}{RT}}. \end{array}$$

As expected in a DCM SEPIC converter in open loop [10,11] an equivalent pure resistive input impedance is deduced from the input current expression.

(11) $$Z_{in}=\frac{{\bar{v}}_{g}}{{\bar{i}}_{L_{1}}}=R_{in}=\frac{2L_{E}}{Td_{1}^{2}}.$$

### 2.4. Generalized Model

#### 2.4.1. Full-Order Dynamic Model

The non-linear model of average values of the SEPIC converter established in (3) can be extended to the Ćuk and Zeta topologies, changing the slope values according to Table 2. Furthermore, because of differences in the output section of the converters, it is necessary to include in the ${\bar{v}}_{C_{2}}$’s equation the variable ς that depends on the converter type to unify the general model. To complete the generalized nonlinear model $d_{2}$ and ${\bar{i}}_{D}$ equations must be included. The resulting model is given in (12).

(12) $$\begin{array}{cc} \frac{d}{dt}{\bar{i}}_{L_{1}} & =m_{11}d_{1}+m_{21}d_{2}+m_{3}\left(1-d_{1}-d_{2}\right)\\ \frac{d}{dt}{\bar{i}}_{L_{2}} & =-\left(m_{12}d_{1}+m_{22}d_{2}+m_{3}\left(1-d_{1}-d_{2}\right)\right)\\ \frac{d}{dt}{\bar{v}}_{C_{1}} & =\frac{1}{C_{1}}\left({\bar{i}}_{D}-{\bar{i}}_{L_{2}}-\frac{1}{R_{d}}\left({\bar{v}}_{C_{1}}-{\bar{v}}_{C_{d}}\right)\right)\\ \frac{d}{dt}{\bar{v}}_{C_{2}} & =\frac{1}{C_{2}}\left(\varsigma {\bar{i}}_{D}+\left(1-\varsigma \right){\bar{i}}_{L_{2}}-\frac{{\bar{v}}_{C_{2}}}{R}\right)\\ \frac{d}{dt}{\bar{v}}_{C_{d}} & =\frac{1}{R_{d}C_{d}}\left({\bar{v}}_{C_{1}}-{\bar{v}}_{C_{d}}\right)\\ d_{2} & =\frac{2({\bar{i}}_{L_{1}}+{\bar{i}}_{L_{2}})}{\left(m_{11}-m_{12}\right)d_{1}T}-d_{1}\\ {\bar{i}}_{D} & =\frac{1}{2}\left(m_{11}-m_{12}\right)d_{1}d_{2}T, \end{array}$$

where ς is a function of the converter:

(13) $$\varsigma =\left\{\begin{array}{cc} \;1;\; & \mathrm{SEPIC}\\ 0; & Ć\mathrm{uk},\;\mathrm{Zeta}.\; \end{array}\right.$$

Note that variables $d_{2}$ and ${\bar{i}}_{D}$ have the same expressions for the three converters: SEPIC, Ćuk and Zeta.

#### 2.4.2. Steady State Operation Point

The methodology to obtain the steady state operating point of the SEPIC converter is valid for all three converters. Equations of ${\bar{i}}_{L_{1}},\;{\bar{i}}_{L_{2}}\;{\bar{v}}_{C_{2}}$ and ${\bar{v}}_{C_{d}}$ shown in (9) are equivalent for Ćuk and Zeta topologies. The only difference is for ${\bar{v}}_{C_{1}}$. Additionally, $d_{2}$ included in (10) is valid for any of the three converters. The generalized expressions of the operating point are given in (14) where having a pure resistive input impedance in terms of average voltage and current is a common characteristic of the SEPIC, Ćuk and Zeta converters in DCM. Note that the input current of the Zeta converter has the same average value than its $L_{1}$ inductor current.

(14) $$\begin{array}{cc} {\bar{i}}_{L_{1}} & =\frac{{\bar{v}}_{g}T}{2L_{E}}d_{1}^{2}\\ {\bar{i}}_{L_{2}} & ={\bar{i}}_{D}=\frac{{\bar{v}}_{g}}{R}\left(\frac{d_{1}}{d_{2}}\right)\\ {\bar{v}}_{C_{1}} & =\left\{\begin{array}{cc} {\bar{v}}_{g}, & \mathrm{SEPIC}\\ {\bar{v}}_{g}+{\bar{v}}_{C_{2}}, & Ć\mathrm{uk}\\ {\bar{v}}_{C_{2}}, & \mathrm{Zeta} \end{array}\right.\\ {\bar{v}}_{C_{2}} & =R{\bar{i}}_{L_{2}}={\bar{v}}_{g}\left(\frac{d_{1}}{d_{2}}\right)\\ {\bar{v}}_{C_{d}} & ={\bar{v}}_{C_{1}}={\bar{v}}_{g}\\ d_{2} & =\sqrt{\frac{2L_{E}}{RT}} \end{array}$$

Note that Equation (11) is valid for all three converters.

#### 2.4.3. Boundary between Continuous and Discontinuous Conduction Mode

Continuous and discontinuous operation modes can be defined according to constant $k=\frac{2L_{E}}{RT}$ as,

(15) $$\left\{\begin{array}{cc} \mathrm{if}\;k>k_{c}, & \mathrm{continuos}\;\mathrm{conduction}\;\mathrm{mode}\;\left(\mathrm{CCM}\right),\\ \mathrm{if}\;k<k_{c}, & \mathrm{discontinuos}\;\mathrm{conduction}\;\mathrm{mode}\;\left(\mathrm{DCM}\right) \end{array}\right.$$

where $k_{c}$ is the *k* value in the boundary between operation modes. It can be easily verified that

(16) $$d_{2}=\sqrt{k}.$$

By replacing this result in the boundary condition $d_{1}=1-d_{2}$ and solving for $k_{c}$, it is obtained

(17) $$k_{c}={\left(1-d_{1}\right)}^{2},$$

which corresponds exactly with the expression of $k_{c}$ presented in the literature for buck-boost converters [2].

**Figure 5:**
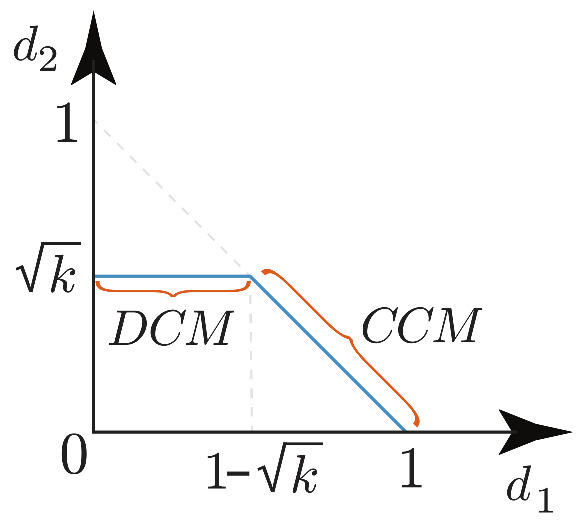
Operation zones of high-order converters.

Furthermore, if the load resistance is constant then $d_{2}$ is also a constant in DCM, while $d_{2}$ satisfies the equation $d_{1}+d_{2}=1$ in CCM. Based on these features it is possible to find the relation between $d_{1}$ and $d_{2}$ shown in Figure 5. The boundary between both modes is the common point between the lines $d_{2}=\sqrt{k}$ and $d_{1}+d_{2}=1$.

#### 2.4.4. Linearized Model

The linearization procedure is well known. The full-order nonlinear model of any of the three converters can be represented as

(18) $$\dot{x}=Ax+Bu.$$

where x is the state vector and u is the input vector defined as $x=\;{\left({\bar{i}}_{L_{1}}\;{\bar{i}}_{L_{2}}\;{\bar{v}}_{C_{1}}\;{\bar{v}}_{C_{2}}\;{\bar{v}}_{C_{d}}\right)}^{T}$ and $u\;={\left(d_{1}\;{\bar{v}}_{g}\right)}^{T}$. To obtain the small signal model, the non-linear model is linearized around an operating point ($x_{o}$). The small signal model can be expressed as

(19) $$\hat{\dot{x}}=A_{o}\hat{x}+B_{o}\hat{u},$$

where $A_{o}$ and $B_{o}$ defined in (20) are the Jacobian matrices evaluated at the operating point.

(20) $$A_{o}=\frac{\partial f}{\partial \bar{x}}{|}_{\bar{x}=x_{o}},\;B_{o}=\frac{\partial f}{\partial u}{|}_{\bar{x}=x_{o}}.$$

Analytical expressions of the small signal model are very complex, therefore it is usually more practical to obtain numerical expressions for each particular case.

## 3. Results

Comparisons between the theoretical and the switched models in time and frequency domains are presented in this section by means of PSIM simulations, hardware-in-the-loop (HIL) tests and experimental results. A description of the nomenclature used is presented below:

- Theoretical results: these are results in the time or frequency domains obtained from the transfer functions of the small-signal models;
- Switched results: they are obtained from simulations in the PSIM software;
- Hardware-in-the-loop results: measurements carried out on the hardware-in-the-loop tools (PLECS RT-box 1, Interface and Texas Instruments LAUNCHXL-F28069M);
- Experimental results: direct measurements in a real proof-of-concept reconfigurable prototype of high-order converters.

In order to explore the scope of the proposed theoretical model, the three high-order converter structures have been used with different magnetic couplings: null, positive and negative. A base set of circuit parameters has been proposed for all three converters. Some parameters were intentionally changed to analyze the response under non-fulfillment of design criteria (high voltage ripple capacitors or inductor currents without triangular waveform).

It is relevant to clarify the notation used to present the results. Until now in this paper, average values nomenclature has been used to express variables from the theoretical model according to equation $\bar{x}=X+\hat{x}$; where $\bar{x}$ is an average value, *X* is a steady state value, and $\hat{x}$ is the small signal value. Thereby ${\bar{i}}_{L_{1}}$, ${\bar{i}}_{L_{2}}$, ${\bar{v}}_{C_{1}}$, ${\bar{v}}_{C_{2}}$ represent theoretical model variables and $i_{L_{1}}$, $i_{L_{2}}$, $v_{C_{1}}$, $v_{C_{2}}$ will be used to denote switched model variables.

### 3.1. Component Description

The selection criteria for the inductors is to keep a fixed value of the self inductance with or without magnetic coupling. The value of $L_{1}$ and $L_{2}$ have been selected equal to 56.4μH. Additionally, the inductors selection fulfills a compromise between a high operating margin in DCM and an acceptable current ripple. A mutual inductance of 47.4μH is achieved with the chosen inductors array, so a coupling factor of $k_{a}=0.84$ is obtained. The mutual inductance can be considered positive or negative depending on the component ports connection. In some cases it has been considered that there is no coupling between $L_{1}$ and $L_{2}$ (M=0).

The values of the capacitors $C_{1}$ and $C_{2}$ were selected considering the criteria of reduced size and approximately constant average voltage. Capacitor values of $C_{1}=\;C_{2}=\;5.0\;\mu$F ensure peak-to-peak ripple capacitor voltage amplitudes less than 4%. Regarding the damping network, its values have been selected, taking into account the guidelines provided in [32]. A capacitor ten times larger than the intermediate one has been selected while the series resistor has been adjusted by simulation so that it ensures a sufficient damping of the internal dynamics. The selected values were 1.5Ω for $R_{d}$ and 50μF for $C_{d}$. The load resistance has also been selected as R=100Ω to ensure DCM at the operation points considered in the study.

Three parameter sets have been defined for the proposed model validation. They are summarized in Table 3. Because of a temporary unavailability of the initially selected loosely-coupled magnetic components [31], an almost equivalent arrangement using Coilcraft’s Hexa-Path perfectly magnetic coupled inductors and other non-coupled inductors has been used. A description of each arrangement is given below:

- Test-1: Base set formed by coupled inductors $L_{1}$, $L_{2}$ with mutual inductance *M*, capacitors $C_{1}$ and $C_{2}$ and a load resistor *R*;
- Test-2: Corresponds with Test-1 but with damping network;
- Test-3: Corresponds with Test-1 with an intermediate capacitor reduced ten times. This test has been defined to study the proposed model under a high voltage ripple condition.

### 3.2. Operation Points

Taking into account the three high-order converter structures and the three possible types of coupling (positive, negative or zero), three case studies have been defined to verify the performance of the proposed full-order model in a broad enough way. The case studies are: Ćuk without magnetic coupling between the inductors, SEPIC with positive magnetic coupling and Zeta with negative magnetic coupling. The operating points listed in Table 4 are obtained by replacing in the theoretical expressions the base set of circuit parameters (Test-1) and the simulation parameters ($V_{g}=\;10$ V, $d_{1}=\;0.4$, $f_{s}=\;100$ kHz). In this table the constant (*k*) allows determining the conduction mode in which the converter operates. This value is less than $k_{crit}={\left(1-d_{1}\right)}^{2}=0.36$, and therefore the converters operate in DCM, as it is verified for all presented cases. Finally, it is satisfied that $v_{C_{d}}=\;v_{C_{1}}$ in steady state.

### 3.3. Transfer Functions

Table 5, Table 6 and Table 7 includes the theoretical transfer functions of inductor currents and capacitor voltages that have been considered in time and frequency domain analysis. In this work, transfer functions can be a function of the input voltage or the duty cycle. The transfer functions can be of 4 or 5 order depending on the addition of the damping network in the topologies. In Table 5, Table 6 and Table 7, it can be seen that the transfer function of $i_{L_{1}}/v_{g}$ is the only one that has all its zeros in the left half-plane in all cases. The other transfer functions have at least one zero in the right half-plane. Another important finding is seen in the analysis of the damping network effect. In the absence of the damping network, the transfer function presents a pair of complex poles with a very small real part 32.48 and very little damped. In fact, they are the dominant poles. With a damping network, the effect of these poles is well damped and the dominant pole becomes a real pole with value of 4012. From the point of view of pole analysis, there is a good damping.

### 3.4. Time Domain Responses

#### 3.4.1. Small Signal Response

**Figure 6:**
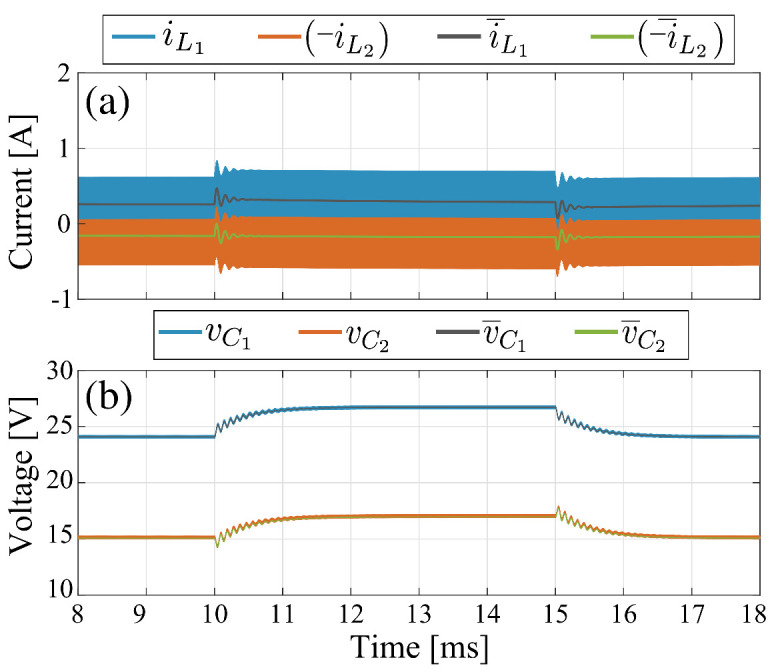
Comparison results in Ćuk converter without coupled inductors: (**a**) inductor currents, (**b**) capacitor voltages.

The objective in small signal is to verify the correspondence between switched and theoretical model responses. The structure used in this comparison is the Ćuk converter without magnetic coupling with the parameter set for the power circuit Test-1. To obtain the results depicted in Figure 6, a reference change in the input voltage of 1 V has been applied. The input voltage $v_{g}$ changes from 9 V to 10 V at t=10 ms and decreases again to 9 V at t=15 ms. The theoretical model results correspond to a linearized model for the operating point $v_{g}=10$ V. In Figure 6a, the averaged model currents have a constant behavior, except during transitions where a small disturbance occurs, but the steady state condition is quickly reached. In Figure 6b it can be observed that the average voltages of the proposed model follow the behavior of the switched model even after the disturbance. The magnitudes obtained by the theoretical model for $v_{g}=10$ V are approximately equal to the operating point values listed in Table 4. Currents and voltages averaged values of both switched and theoretical model for $v_{g}=9$ V and $v_{g}=10$ V are given in Table 8. These results validate the good performance of the theoretical model calculated for $v_{g}=10$ V condition to approximate the average values of the responses obtained from the switched simulations for $v_{g}=9$ V and $v_{g}=10$ V. The relative error (RE) were less than 0.51% in all cases. The results show that the $v_{g}=9$ V condition is a small signal operation as had been supposed before.

#### 3.4.2. Damping Network Effect

**Figure 7:**
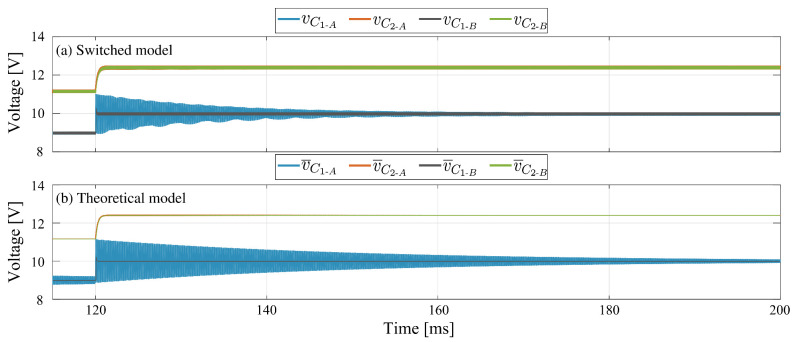
Comparison results for capacitor voltages in SEPIC converter with positive magnetic coupling, (A) without damping network, (B) with damping network: (**a**) switched model, (**b**) theoretical model.

A damping network can be used in parallel with the intermediate capacitor to decrease the settling time. In the analysis carried out in this work, the SEPIC converter was the topology that, without a damping network, requires the longest time to reach a steady state. For this reason, the positive-coupled SEPIC converter was used specifically to analyze the damping network effect on the capacitors voltage. Two parameter sets have been used in the simulation: Test-1 for response without damping network and Test-2 for the analysis of the damping network effect. Switched and theoretical model responses are depicted in Figure 7a,b. In the presented results, the input voltage changes from 9 V to 10 V at time t=120 ms. The direct effect of the damping network is observed in the intermediate capacitor voltage. In the absence of the damping network, the voltage requires a significant settling time (in the order of 80 ms) to reach steady state. On the other hand, with the addition of the damping network, the settling time is minimal. These behaviors are equivalent in both switched and theoretical models. It can be verified in graphs from Figure 7 that the theoretical model of average values also has an equivalent performance to the switched model when the damping network is added to the power circuit of the higher-order converter.

#### 3.4.3. Non-Fulfillment of Design Criteria

**Figure 8:**
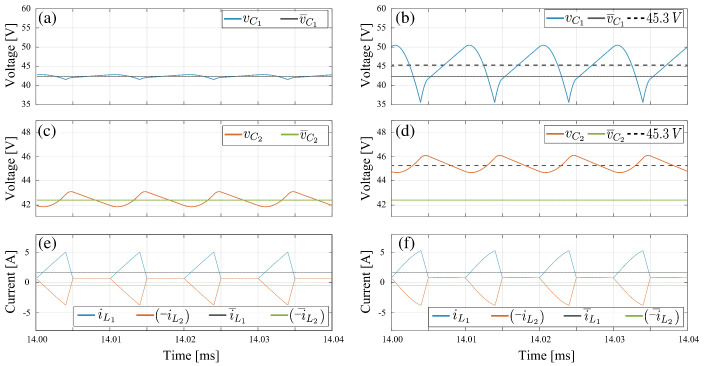
Comparison results for capacitor voltages in Zeta converter with negative magnetic coupling: (**a**,**b**) intermediate capacitor voltage ($v_{C_{1}}$), (**c**,**d**) output capacitor voltage ($v_{C_{2}}$), (**e**,**f**) inductor current, (**a**,**c**,**e**) $C_{1}=5$μF, (**b**,**d**,**f**)$C_{1}=0.5$μF.

The topology considered in this case is the negative-coupled Zeta converter. The purpose of this analysis is to show that, when the low ripple condition is not fulfilled in the capacitors, the theoretical averaged model does not approximate accurately the switched model. In this section, the Test-1 and Test-3 parameter sets have been considered. The difference is that the value of $C_{1}$ passed from 5μF in Test-1 to 0.5μF in Test-3. Derating the capacitor by a factor of 10 times is a violation of the design criteria of approximately constant capacitor voltage. Note that in the Zeta converter the averaged voltages in $C_{1}$ and $C_{2}$ are equal. According to Table 4 this value is 42.16 V. It can be verified in Figure 8 that ${\bar{v}}_{C_{1}}$ and ${\bar{v}}_{C_{2}}$ are equal to the theoretical value in all cases, since the operation point in steady state does not depend of the capacitor values. It is also observed that for the condition $C_{1}=5\;\mu$F the average values of the switched model voltages are approximately equal to the theoretical model values. When the capacitor $C_{1}$ changes to 0.5μF two situations are evident: (i) the voltage ripple in $C_{1}$ increases from 3.1% under design conditions to 34%, while the ripple of $C_{2}$ remains constant, (ii) the average values voltages in $C_{1}$ and $C_{2}$ for the switched model increase and differ from the theoretical model values.

In Figure 8d, it is observed that ${\bar{v}}_{C_{2}}$ does not correspond to the average value of $v_{C_{2}}$. It can also be easily verified by area analysis in Figure 8d, that ${\bar{v}}_{C_{1}}$ does not correspond to the average value of $v_{C_{1}}$. In fact the new average value of $v_{C_{1}}$ and $v_{C_{2}}$ is 45.3 V. It is concluded that the theoretical model values do not correspond with the average value of the switched model, which increased by 3.1 V with the decrement in $C_{1}$ capacitor. An important comment on the currents comparison in Figure 8 is about their waveform shapes. In Figure 8e both currents, $i_{L_{1}}$ and $i_{L_{2}}$, have a piecewise linear function of triangular shape while in Figure 8f, the triangular waveform is not completely linear. Logically the capacitors ripple increment affects the currents triangular shape, which also demonstrates that in the high voltage ripple condition the theoretical model does not faithfully reproduce the switched model results.

#### 3.4.4. HIL Validation

**Figure 9:**
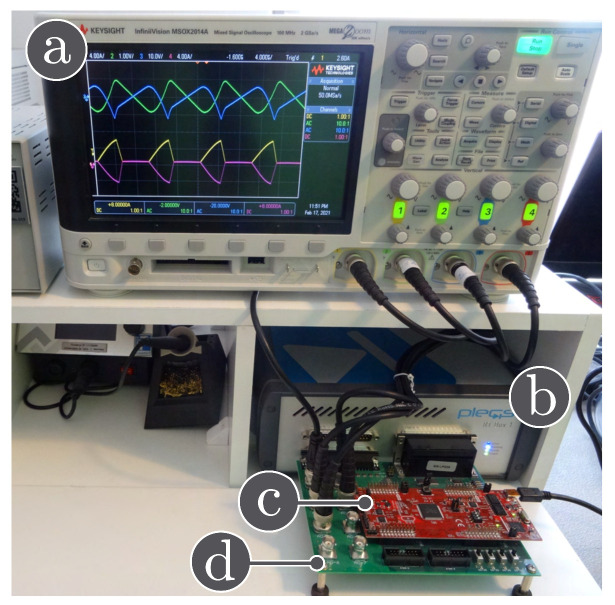
Hardware-in-the-loop experimental setup: (**a**) oscilloscope, (**b**) PLECS RT-box 1, (**c**) Texas Instruments LAUNCHXL-F28069M, (**d**) RT Box LaunchPad Interface.

Nowadays, the use of Hardware-in-the-loop (HIL) tools to validate the controllers performance is more popular [33]. In this paper, different tests have been carried out in HIL to validate the simulation results in the high-order switched DC-DC converters previously presented by means of the experimental setup shown in Figure 9. The HIL testing system consists of:

- A TI 28069M LaunchPad;
- An RT Box LaunchPad Interface;
- A laptop with the PLECS software;
- An oscilloscope Keysight MSOX2014A,

where the evaluation kit, a TI 28069M LaunchPad (the red board), is connected to the RT Box via an RT Box LaunchPad Interface (the green board). The differente high-order switched DC-DC converters has been modelled using PLECS RT Box 1. In this way, the converter duty cycle has been generated using TI 28069M LaunchPad, which is a Texas Instrument microcontroller.

**Figure 10:**
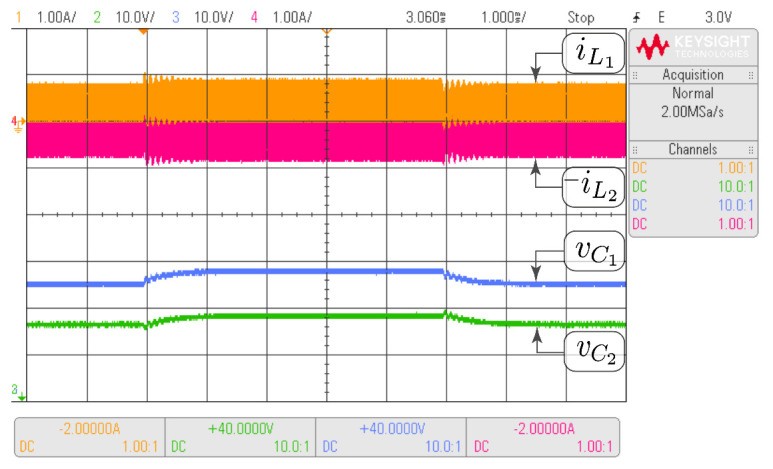
HIL test to validate the proposed model and the simulation of the switched model using PSIM by the Ćuk converter without coupled inductors shown in Figure 6. CH1: $i_{l_{1}}$ (1 A/div), CH2: $v_{C_{2}}$ (10 V/div), CH3: $v_{C_{1}}$ (10 V/div), CH4: $-i_{l_{2}}$ (1 A/div), and time base of 1 ms.

**Figure 11:**
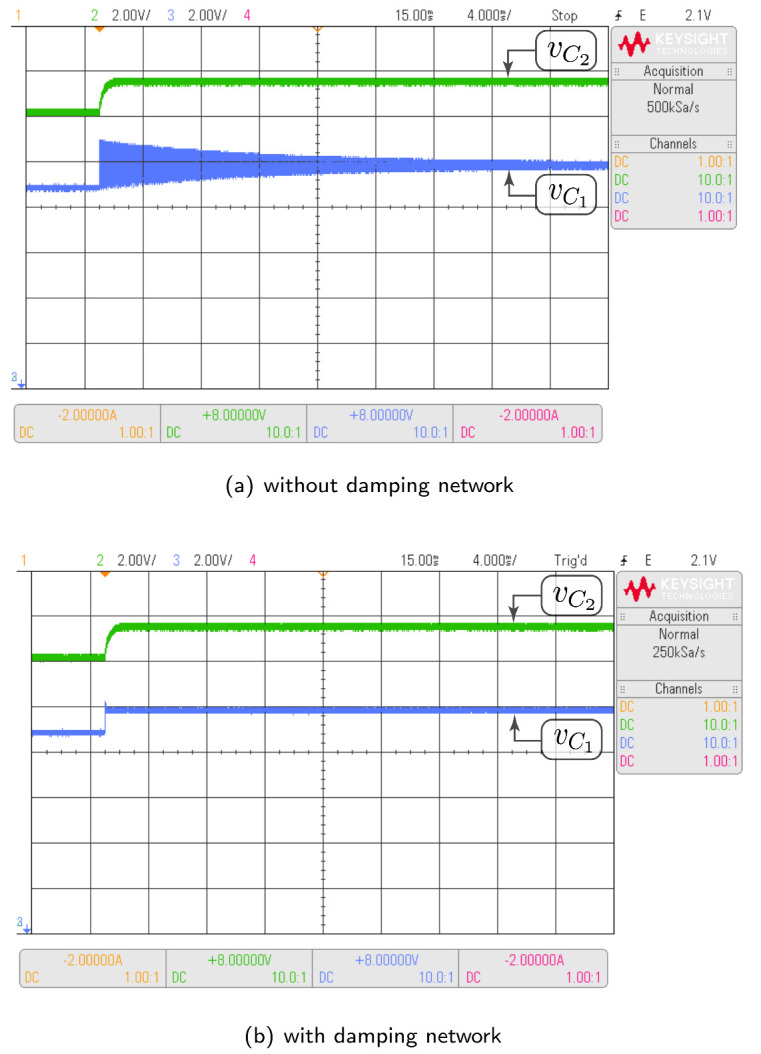
HIL test to validate the proposed model and the simulation of the switched model using PSIM by the SEPIC converter with positive magnetic coupling shown in Figure 7: (**a**) without damping network, (**b**) with damping network. (CH2: $v_{C_{2}}$ (2 V/div), CH3: $v_{C_{1}}$ (2 V/div), and time base of 4 ms).

**Figure 12:**
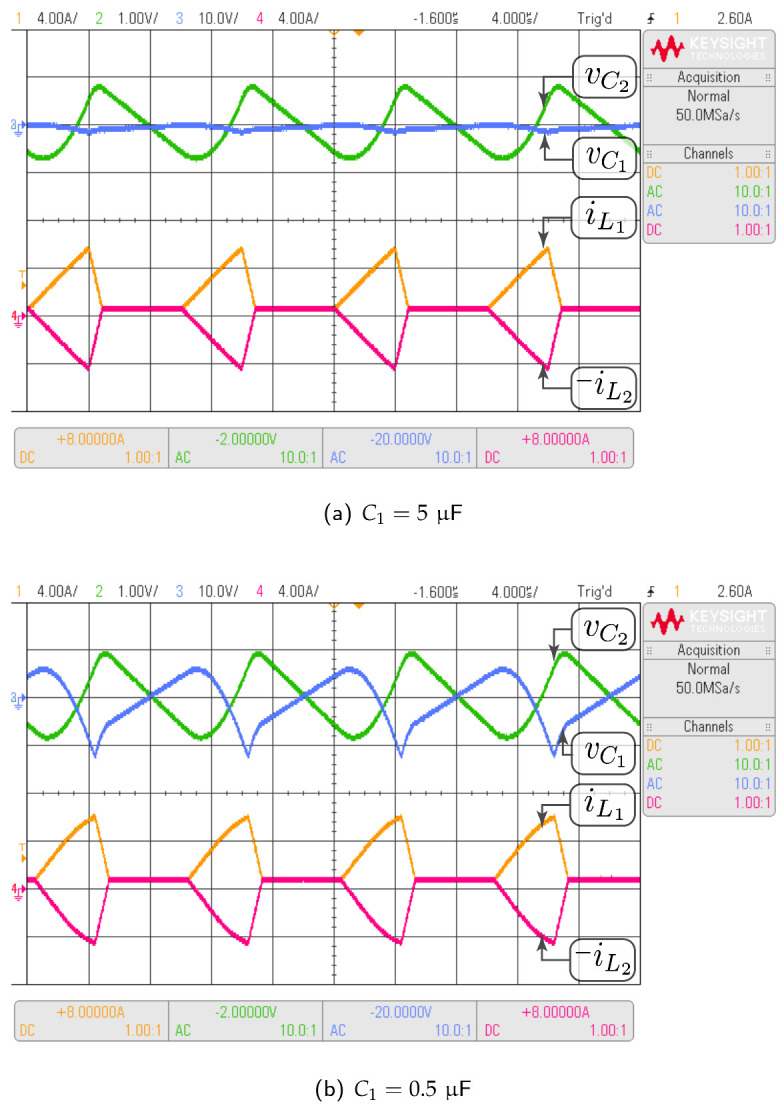
HIL test to validate the proposed model and the simulation of the switched model using PSIM by the Zeta converter with negative magnetic coupling shown in Figure 8: (**a**) $C_{1}=5$μF, (**b**) $C_{1}=0.5$μF. CH1: $i_{l_{1}}$ (4 A/div), CH2: $v_{C_{2}}$ (1 V/div, ac coupling), CH3: $v_{C_{1}}$ (10 V/div, ac coupling), CH4: $-i_{l_{2}}$ (4 A/div), and time base of 4 ms.

In this subsection, the HIL test is presented with the goal of validating the time domain responses presented in the previous subsections. Figure 10 shows the HIL test to compare the proposed model and the simulation of the switched model using PSIM of the Ćuk converter without coupled inductors shown in Figure 6. In addition, the HIL test to validate the proposed model and the simulation of the switched model using PSIM of the SEPIC converter with positive magnetic coupling shown in Figure 7 is presented in Figure 11. Finally, the proposed model and simulation in Figure 8 correspond with the the HIL test of the Zeta converter with negative magnetic coupling shown in Figure 12. A good agreement between the model, the switching simulations and the HIL results is observed in all the cases. Therefore, the proposed procedure models correctly the considered high order step-up and down converter topologies (SEPIC, Ćuk or Zeta) operating in DCM.

#### 3.4.5. Experimental Results

**Figure 13:**
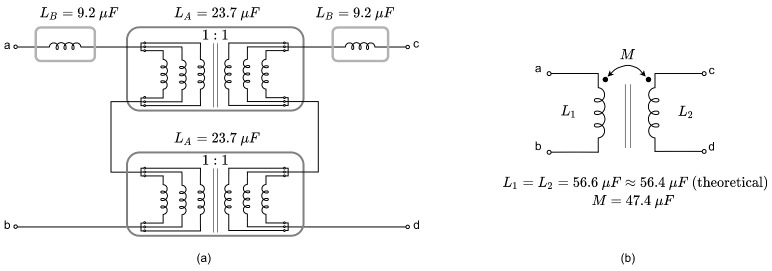
Arrangement to obtain the inductors with magnetic coupling: (**a**) interconnections between inductors, (**b**) equivalent coupled inductors.

A reconfigurable power converter was built to validate the theoretical models and the HIL results. Its design allows the implementation of any topology and any magnetic coupling. The components description of the power converter is presented in Table 9. Considering the reconfigurable characteristic of the power converter, series and parallel interconnections between inductors or capacitors have been done to obtain the closest values to the parameters listed in Table 3. The components configurations are given below:

- Coupled inductors: the arrangement to obtain the inductors with magnetic coupling consist of two perfectly magnetic coupled inductors of 9.2 μH with two external inductors of 23.7 μH. The result is two coupled inductors with a mutual inductance of 47.4 μH and equal self inductance of 56.6 μH. The inductors arrangement and its equivalent circuit are shown in Figure 13;
- Non-coupled inductors: it is a series arrangement of inductors of 47 μH and 9.2 μH;
- Intermediate capacitor 0.5 μF: 5 capacitors of 100 nF connected in parallel;
- Intermediate capacitor 5 μF: 2 capacitors of 10 μF connected in series;
- Damping network capacitor 50 μF: 5 capacitors of 10 μF connected in parallel.

**Figure 14:**
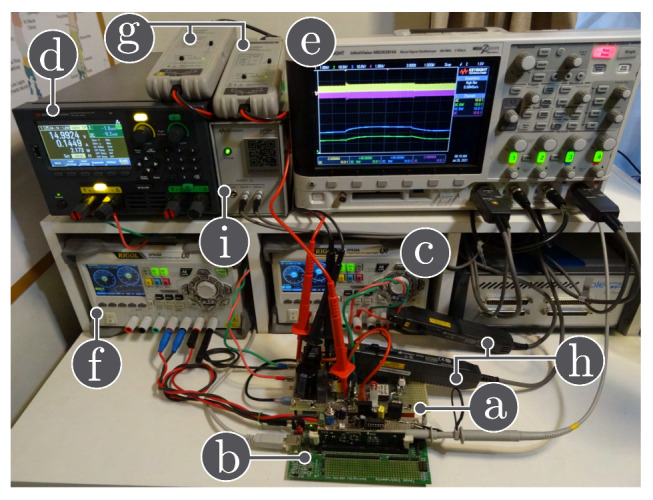
Experimental setup: (**a**) reconfigurable power converter, (**b**) digital signal controller, (**c**) input dc power supply, (**d**) dc electronic load in constant resistance mode of 100Ω, (**e**) oscilloscope, (**f**) auxiliary power supply, (**g**) voltage differential probes, (**h**) current probes, (**i**) power supply for the current probes.

**Figure 15:**
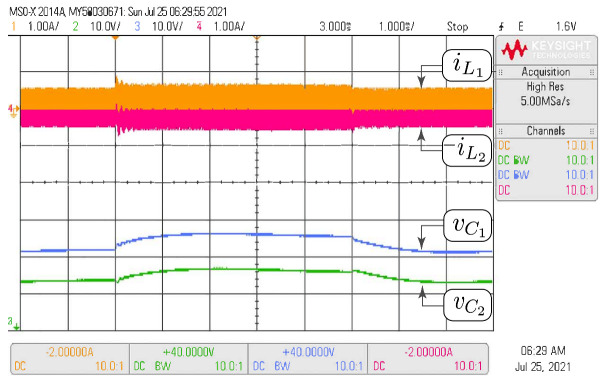
Experimental results for the Ćuk converter without coupled inductors which validate HIL results showed in Figure 10. CH1: $i_{l_{1}}$ (1 A/div), CH2: $v_{C_{2}}$ (10 V/div), CH3: $v_{C_{1}}$ (10 V/div), CH4: $-i_{l_{2}}$ (1 A/div), and time base of 1 ms.

**Figure 16:**
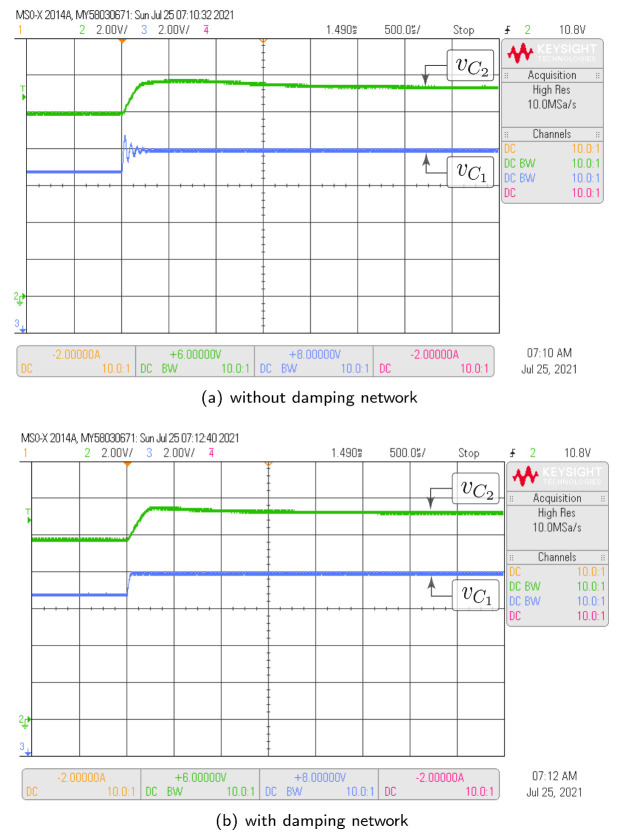
Experimental results for the SEPIC converter with positive magnetic coupling which demonstrate good agreement with HIL results showed in Figure 11: (**a**) without damping network, (**b**) with damping network. (CH2: $v_{C_{2}}$ (2 V/div), CH3: $v_{C_{1}}$ (2 V/div), and time base of 500 μs).

**Figure 17:**
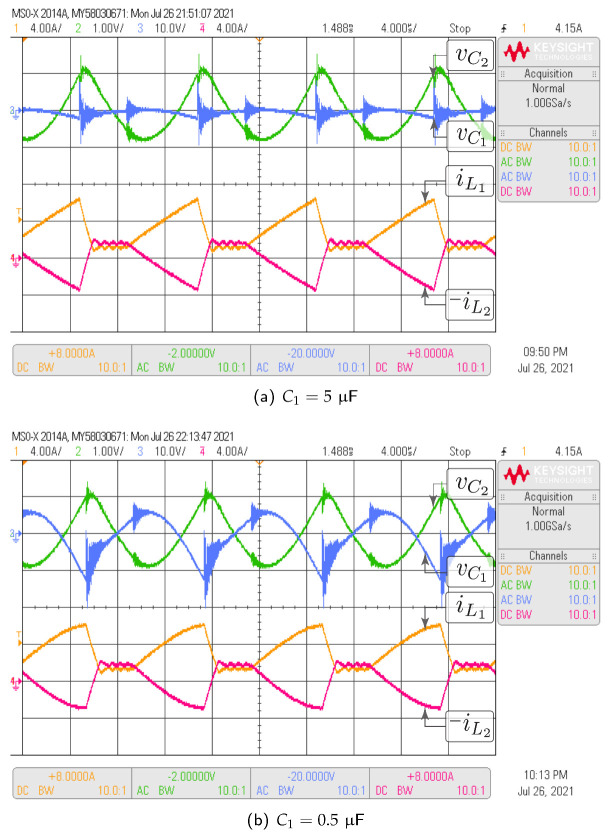
Experimental results for the Zeta converter with negative magnetic coupling which validate HIL results showed in Figure 12: (**a**) $C_{1}=5$μF, (**b**) $C_{1}=0.5$μF. CH1: $i_{l_{1}}$ (4 A/div), CH2: $v_{C_{2}}$ (1 V/div, ac coupling), CH3: $v_{C_{1}}$ (10 V/div, ac coupling), CH4: $-i_{l_{2}}$ (4 A/div), and time base of 4 μs.

The same tests carried out with HIL have been performed with the converter prototype. The experimental setup of the power circuit is shown in Figure 14. Voltage and current waveforms for a Ćuk converter without magnetic coupling are shown in Figure 15. The effect of the damping network in the SEPIC converter with positive magnetic coupling is shown in Figure 16. Finally, the results of the Zeta converter with negative magnetic coupling are shown in Figure 17. The waveforms in Figure 15 and Figure 16 show good agreement with waveforms in Figure 10 and Figure 11. The results shown in Figure 17 have some distortion due to resonances with parasitic capacitances, but they are similar to the ideal waveforms shown in Figure 12.

### 3.5. Frequency Domain Responses

**Figure 18:**
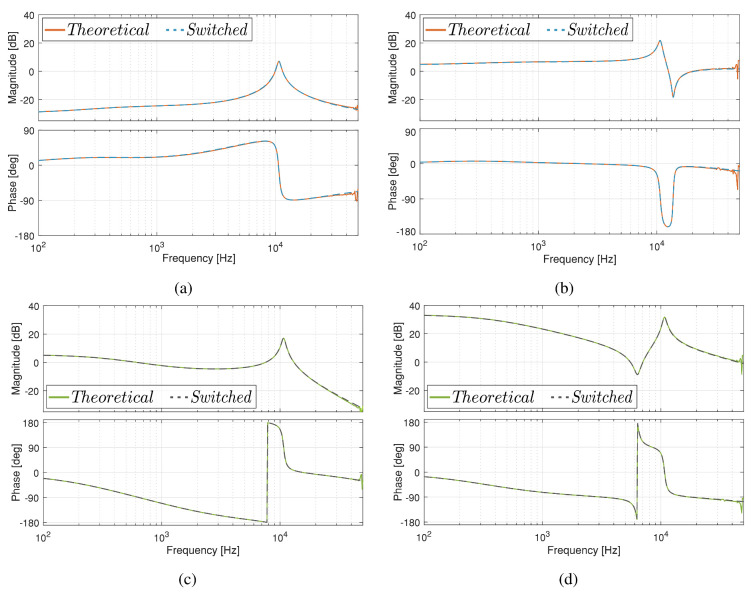
Frequency response of theoretical and switched models for: (**a**) $\frac{{\hat{i}}_{L_{1}}\left(s\right)}{{\hat{v}}_{g}\left(s\right)}$, (**b**) $\frac{{\hat{i}}_{L_{1}}\left(s\right)}{\hat{d}\left(s\right)}$, (**c**) $\frac{{\hat{v}}_{C_{2}}\left(s\right)}{{\hat{v}}_{g}\left(s\right)}$, and (**d**) $\frac{{\hat{v}}_{C_{2}}\left(s\right)}{\hat{d}\left(s\right)}$.

The Ćuk converter without magnetic coupling has been used to analyze the theoretical model’s performance. The same topology was used in the small signal validation. The variables selected for this analysis are one current and one voltage, specifically $i_{L_{1}}$ and $v_{C_{2}}$. To obtain broad information on the frequency domain response, transfer functions were considered a function of the input voltage and the duty cycle, which are included in Table 5. The lower limit of the frequency analysis is 100 Hz and the upper limit is 50 kHz, a value that corresponds to half the switching frequency. It is observed in all the bode plots shown in Figure 18 that the theoretical model is equivalent to the switched model response. Another common feature is a resonance that occurs at approximately 10 kHz.

### 3.6. Frequency Domain Validation

**Figure 19:**
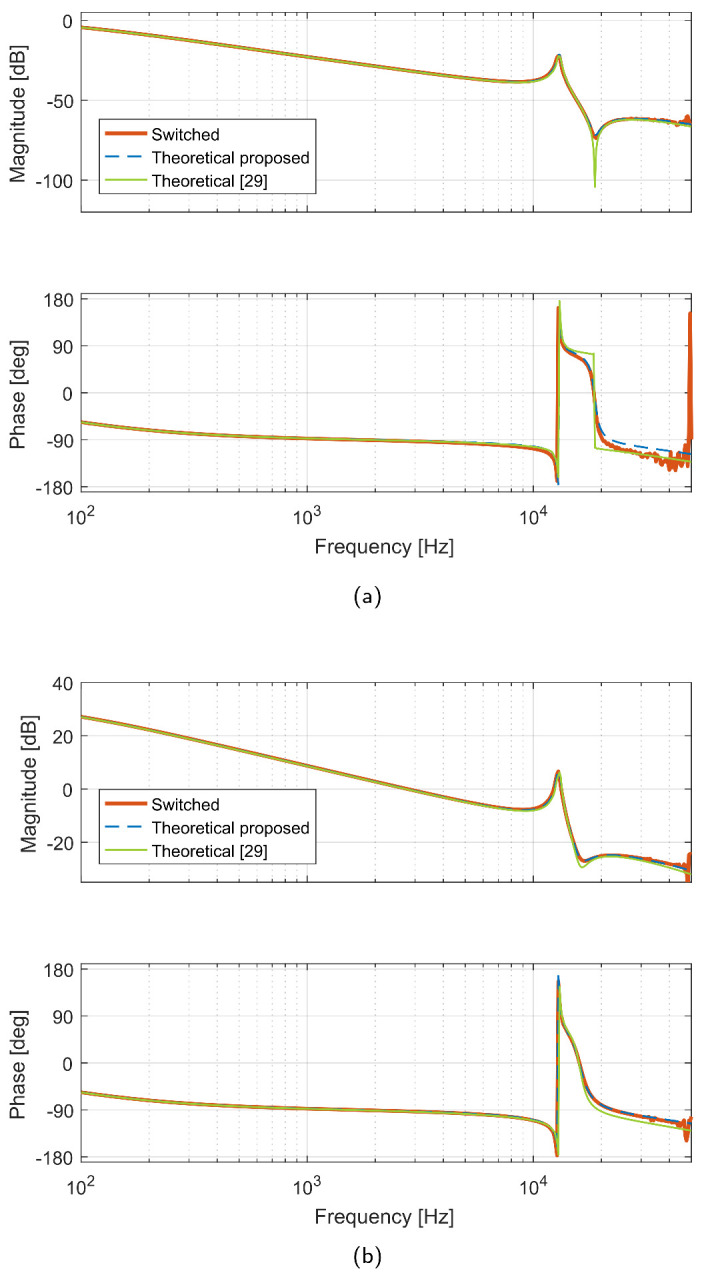
Frequency response comparison: (**a**) $\frac{{\hat{v}}_{C_{2}}\left(s\right)}{{\hat{v}}_{g}\left(s\right)}$, (**b**) $\frac{{\hat{v}}_{C_{2}}\left(s\right)}{\hat{d}\left(s\right)}$.

Another way to validate the good performance of the proposed methodology is by comparing results with the generalized switch averaging technique [6] in a specific case using frequency responses. To make a fair comparison with the literature, a full-order dynamic model of a Zeta converter in DCM presented in [29] was chosen. The power circuit parameters used to calculate the transfer functions and to obtain the frequency responses are the same as those proposed in [29]. The transfer functions obtained with both methodologies are given in Table 10. Note that all of the models are fourth order transfer functions and the numerator polynomial is second order. The corresponding frequency responses are shown in Figure 19. Both theoretical responses show a good agreement of the frequency response of the switched model up to a frequency about 1/5 of the switching one. Note that frequency response of reduced-order models is usually considered accurate up to about 1/20–1/10 of the switching frequency.

## 4. Conclusions

In this paper, a methodology for analyzing high-order switched converters, specifically SEPIC, Ćuk and Zeta, operating in discontinuous conduction mode, is proposed to obtain a full-order dynamic model. The studied converters have very similar piecewise linear current functions if small capacitor voltage ripples are assumed. These converters present a special discontinuity mode where the inductor currents are constant and different from zero. This behavior occurs at the third sub-interval of the switching period in which neither the MOSFET nor the diode conduct. On the basis of the triangular shape of the current diode non-linear average models are obtained. The methodology can be applied to any high-order converter even if additional attributes, such as magnetic coupling or a damping network, are considered. A generalized full-order dynamic model and the steady state operation point summarize the theoretical analysis. Simulations, HIL tests and experimental results validated some full-order small signal models. The comparison of the results demonstrate that theoretical small signal models accurately reproduce the switched models around the operation points as long as the low ripple condition is satisfied. Future research will deepen in the applications, such as power factor correction or visible light communication, the use of the dynamic DCM model to design control laws of the analyzed high-order converters, and the extension of the proposed model’s methodology to other converter topologies not modeled in DCM to date.

## Figures and Tables

**Table 1:** Dynamic model slopes for the SEPIC converter.

Slope	Equation
$m_{11}$	$\frac{L_{2}{\bar{v}}_{g}-M{\bar{v}}_{C_{1}}}{\Delta L}$
$m_{21}$	$\frac{L_{2}\left({\bar{v}}_{g}-\left({\bar{v}}_{C_{1}}+{\bar{v}}_{C_{2}}\right)\right)+M{\bar{v}}_{C_{2}}}{\Delta L}$
$m_{12}$	$\frac{-L_{1}{\bar{v}}_{C_{1}}+M{\bar{v}}_{g}}{\Delta L}$
$m_{22}$	$\frac{L_{1}{\bar{v}}_{C_{2}}+M\left({\bar{v}}_{g}-\left({\bar{v}}_{C_{1}}+{\bar{v}}_{C_{2}}\right)\right)}{\Delta L}$
$m_{3}$	$\frac{{\bar{v}}_{g}-{\bar{v}}_{C_{1}}}{L_{s}}$

with $\Delta L=L_{1}L_{2}-M^{2}$, $L_{s}=L_{1}+L_{2}-2M$.

**Table 2:** Generalized slope of the inductor current waveform in the SEPIC, Ćuk and Zeta converters.

Slope	SEPIC	Ćuk	Zeta
$m_{11}$	$\frac{L_{2}{\bar{v}}_{g}-M{\bar{v}}_{C_{1}}}{\Delta L}$	$\frac{L_{2}{\bar{v}}_{g}-M\left({\bar{v}}_{C_{1}}-{\bar{v}}_{C_{2}}\right)}{\Delta L}$	$\frac{L_{2}{\bar{v}}_{g}-M\left({\bar{v}}_{g}+{\bar{v}}_{C_{1}}-{\bar{v}}_{C_{2}}\right)}{\Delta L}$
$m_{21}$	$\frac{L_{2}\left({\bar{v}}_{g}-\left({\bar{v}}_{C_{1}}+{\bar{v}}_{C_{2}}\right)\right)+M{\bar{v}}_{C_{2}}}{\Delta L}$	$\frac{L_{2}\left({\bar{v}}_{g}-{\bar{v}}_{C_{1}}\right)+M{\bar{v}}_{C_{2}}}{\Delta L}$	$\frac{-L_{2}{\bar{v}}_{C_{1}}+M{\bar{v}}_{C_{2}}}{\Delta L}$
$m_{12}$	$\frac{-L_{1}{\bar{v}}_{C_{1}}+M{\bar{v}}_{g}}{\Delta L}$	$\frac{-L_{1}\left({\bar{v}}_{C_{1}}-{\bar{v}}_{C_{2}}\right)+M{\bar{v}}_{g}}{\Delta L}$	$\frac{-L_{1}\left({\bar{v}}_{g}+{\bar{v}}_{C_{1}}-{\bar{v}}_{C_{2}}\right)+M{\bar{v}}_{g}}{\Delta L}$
$m_{22}$	$\frac{L_{1}{\bar{v}}_{C_{2}}+M\left({\bar{v}}_{g}-\left({\bar{v}}_{C_{1}}+{\bar{v}}_{C_{2}}\right)\right)}{\Delta L}$	$\frac{L_{1}{\bar{v}}_{C_{2}}+M\left({\bar{v}}_{g}-{\bar{v}}_{C_{1}}\right)}{\Delta L}$	$\frac{L_{1}{\bar{v}}_{C_{2}}-M{\bar{v}}_{C_{1}}}{\Delta L}$
$m_{3}$	$\frac{{\bar{v}}_{g}-{\bar{v}}_{C_{1}}}{L_{s}}$	$\frac{{\bar{v}}_{g}-{\bar{v}}_{C_{1}}+{\bar{v}}_{C_{2}}}{L_{s}}$	$\frac{{\bar{v}}_{C_{2}}-{\bar{v}}_{C_{1}}}{L_{s}}$.

**Table 3:** Test parameters.

Parameter	Test-1	Test-2	Test-3
$L_{1}$ [μH]	56.4	56.4	56.4
$L_{2}$ [μH]	56.4	56.4	56.4
*M* [μH]	47.4	47.4	47.4
$C_{1}$ [μF]	5.0	5.0	0.5
$C_{2}$ [μF]	5.0	5.0	5.0
$C_{d}$ [μF]	−	50.0	−
$R_{d}$ [Ω]	−	1.5	−
*R* [Ω]	100.0	100.0	100.0

**Table 4:** Theoretical operation points for the Test-1 parameters.

Parameter	Ćuk (M=0)	SEPIC (M>0)	Zeta (M<0)
${\bar{i}}_{L_{1}}$ [A]	0.2837	0.1541	1.7778
${\bar{i}}_{L_{2}}$ [A]	0.1684	0.1242	0.4216
${\bar{v}}_{C_{1}}$ [V]	26.8430	10.0000	42.1637
${\bar{v}}_{C_{2}}$ [V]	16.8430	12.4154	42.1617
$d_{2}$ [-]	0.2375	0.3222	0.0949
$I_{3}$ [A]	0.0596	0.01845	0.7095
*k* [-]	0.0564	0.1038	0.0090

**Table 5:** Theoretical transfer functions of the Ćuk converter under the tests conditions depicted in Table 3.

Description	Transfer Function
Ćuk (M=0, Test-1)	$\frac{{\hat{i}}_{L_{1}}\left(s\right)}{{\hat{v}}_{g}\left(s\right)}=\frac{\left(s+1080.26)(s+22366.82)(s+291761.29\right)}{\left(s+2004.87\right)\left(s+841142.14\right)\left({\left(s+1920.90\right)}^{2}+59481.49^{2}\right)}=\frac{N_{1}\left(s\right)}{D_{1}\left(s\right)}$
	$\frac{{\hat{i}}_{L_{1}}\left(s\right)}{\hat{d}\left(s\right)}=\frac{\left(s+1542.11\right)\left({\left(s+1335.31\right)}^{2}+75958.36^{2}\right)}{D_{1}\left(s\right)}$
	$\frac{{\hat{i}}_{L_{2}}\left(s\right)}{{\hat{v}}_{g}\left(s\right)}=\frac{\left(s+2000)(s-6740.96)(s-1183456.26\right)}{D_{1}\left(s\right)}$
	$\frac{{\hat{i}}_{L_{2}}\left(s\right)}{\hat{d}\left(s\right)}=\frac{\left(s+1542.11\right)\left({\left(s+1335.31\right)}^{2}+75958.36^{2}\right)}{D_{1}\left(s\right)}$
	$\frac{{\hat{v}}_{C_{1}}\left(s\right)}{{\hat{v}}_{g}\left(s\right)}=\frac{\left(s+10972.63)(s+406309.64)(s-1267635.58\right)}{D_{1}\left(s\right)}$
	$\frac{{\hat{v}}_{C_{2}}\left(s\right)}{{\hat{v}}_{g}\left(s\right)}=\frac{\left(s-6740.96)(s-1183456.26\right)}{D_{1}\left(s\right)}$
	$\frac{{\hat{v}}_{C_{2}}\left(s\right)}{\hat{d}\left(s\right)}=\frac{\left({\left(s-1535.74\right)}^{2}+36313.79^{2}\right)}{D_{1}\left(s\right)}$
	$\frac{{\hat{v}}_{C_{2}}\left(s\right)}{{\hat{i}}_{L_{1}}\left(s\right)}=\frac{\left({\left(s-1535.74\right)}^{2}+36313.79^{2}\right)}{\left(s+1542.11\right)\left({\left(s+1335.31\right)}^{2}+75958.36^{2}\right)}$
	$\frac{{\hat{v}}_{C_{2}}\left(s\right)}{{\hat{i}}_{L_{2}}\left(s\right)}=\frac{\left({\left(s-1535.74\right)}^{2}+36313.79^{2}\right)}{\left(s+1542.11\right)\left({\left(s+1335.31\right)}^{2}+75958.36^{2}\right)}$
	${\hat{Z}}_{in}\left(s\right)=\frac{D_{1}\left(s\right)}{\left(s+1080.26)(s+22366.82)(s+291761.29\right)}$
	${\hat{Z}}_{out}\left(s\right)=\frac{\left(s+844748.93\right)\left({\left(s+119.93\right)}^{2}+42042.65^{2}\right)}{D_{1}\left(s\right)}$

**Table 6:** Theoretical transfer functions of the SEPIC converter under the tests conditions depicted in Table 3.

Description	Transfer Function
SEPIC (M>0, Test-1)	$\frac{{\hat{i}}_{L_{1}}\left(s\right)}{\hat{d}\left(s\right)}=\frac{\left(s+4011.73\right)\left({\left(s+9394.93\right)}^{2}+110381.85^{2}\right)}{\left(s+4012.47\right)\left(s+620234.85\right)\left({\left(s+32.48\right)}^{2}+105290.84^{2}\right)}=\frac{N_{2}\left(s\right)}{D_{2}\left(s\right)}$
	$\frac{{\hat{i}}_{L_{2}}\left(s\right)}{\hat{d}\left(s\right)}=\frac{\left(s+2000.45\right)\left({\left(s+9427.05\right)}^{2}+99109.72^{2}\right)}{D_{2}\left(s\right)}$
	$\frac{{\hat{v}}_{C_{1}}\left(s\right)}{{\hat{v}}_{g}\left(s\right)}=\frac{\left(s+4014.30)(s+547190.15)(s-8148514.90\right)}{D_{2}\left(s\right)}$
	$\frac{{\hat{v}}_{C_{2}}\left(s\right)}{{\hat{v}}_{g}\left(s\right)}=\frac{\left(s-1398640.41\right)\left({\left(s-2042.34\right)}^{2}+126032.83^{2}\right)}{D_{2}\left(s\right)}$
	$\frac{{\hat{v}}_{C_{2}}\left(s\right)}{\hat{d}\left(s\right)}=\frac{\left(s-499856.40\right)\left({\left(s-71.80\right)}^{2}+105424.37^{2}\right)}{D_{2}\left(s\right)}$
	$\frac{{\hat{v}}_{C_{2}}\left(s\right)}{{\hat{i}}_{L_{1}}\left(s\right)}=\frac{\left(s-499856.40\right)\left({\left(s-71.80\right)}^{2}+105424.37^{2}\right)}{\left(s+4011.73\right)\left({\left(s+9394.93\right)}^{2}+110381.85^{2}\right)}$
	$\frac{{\hat{v}}_{C_{2}}\left(s\right)}{{\hat{i}}_{L_{2}}\left(s\right)}=\frac{\left(s-499856.40\right)\left({\left(s-71.80\right)}^{2}+105424.37^{2}\right)}{\left(s+2000.45\right)\left({\left(s+9427.05\right)}^{2}+99109.72^{2}\right)}$
	${\hat{Z}}_{in}\left(s\right)=\frac{D_{2}\left(s\right)}{\left(s+275.00)(s+4014.35)(s+149581.03)(s+542015.57\right)}$
	${\hat{Z}}_{out}\left(s\right)=\frac{\left(s+622241.00\right)\left({\left(s+35.65\right)}^{2}+105284.65^{2}\right)}{D_{2}\left(s\right)}$
SEPIC (M>0, Test-2)	$\frac{{\hat{i}}_{L_{1}}\left(s\right)}{\hat{d}\left(s\right)}=\frac{\left(s+3995.17\right)\left(s+16523.76\right)\left({\left(s+74474.66\right)}^{2}+66313.01^{2}\right)}{\left(s+4012.47\right)\left(s+16534.61\right)\left(s+620635.63\right)\left({\left(s+64898.11\right)}^{2}+68718.26^{2}\right)}=\frac{N_{3}\left(s\right)}{D_{3}\left(s\right)}$
	$\frac{{\hat{i}}_{L_{2}}\left(s\right)}{\hat{d}\left(s\right)}=\frac{\left(s+2005.69\right)\left(s+16545.91\right)\left({\left(s+55630.71\right)}^{2}+69796.15^{2}\right)}{D_{3}\left(s\right)}$
	$\frac{{\hat{v}}_{C_{1}}\left(s\right)}{{\hat{v}}_{g}\left(s\right)}=\frac{\left(s+4014.30)(s+13333.33)(s+547190.15)(s-8148514.90\right)}{D_{3}\left(s\right)}$
	$\frac{{\hat{v}}_{C_{2}}\left(s\right)}{{\hat{v}}_{g}\left(s\right)}=\frac{\left(s+15256.57\right)\left(s-1398991.10\right)\left({\left(s+63838.05\right)}^{2}+99029.19^{2}\right)}{D_{3}\left(s\right)}$
	$\frac{{\hat{v}}_{C_{2}}\left(s\right)}{\hat{d}\left(s\right)}=\frac{\left(s+16534.58\right)\left(s-499885.01\right)\left({\left(s+65008.55\right)}^{2}+68817.48^{2}\right)}{D_{3}\left(s\right)}$
	$\frac{{\hat{v}}_{C_{2}}\left(s\right)}{{\hat{i}}_{L_{1}}\left(s\right)}=\frac{\left(s+16534.58\right)\left(s-499885.01\right)\left({\left(s+65008.55\right)}^{2}+68817.48^{2}\right)}{\left(s+3995.17\right)\left(s+16523.76\right)\left({\left(s+74474.66\right)}^{2}+66313.01^{2}\right)}$
	$\frac{{\hat{v}}_{C_{2}}\left(s\right)}{{\hat{i}}_{L_{2}}\left(s\right)}=\frac{\left(s+16534.58\right)\left(s-499885.01\right)\left({\left(s+65008.55\right)}^{2}+68817.48^{2}\right)}{\left(s+2005.69\right)\left(s+16545.91\right)\left({\left(s+55630.71\right)}^{2}+69796.15^{2}\right)}$
	${\hat{Z}}_{in}\left(s\right)=\frac{D_{3}\left(s\right)}{\left(s+275.00)(s+4014.35)(s+149581.03)(s+542015.57\right)}$
	${\hat{Z}}_{out}\left(s\right)=\frac{\left(s+16535.58\right)\left(s+622638.33\right)\left({\left(s+64902.52\right)}^{2}+68703.34^{2}\right)}{D_{3}\left(s\right)}$

**Table 7:** Theoretical transfer functions of the Zeta converter under the tests conditions depicted in Table 3.

Description	Transfer Function
Zeta (M<0, Test-1)	$\frac{{\hat{i}}_{L_{1}}\left(s\right)}{{\hat{v}}_{g}\left(s\right)}=\frac{\left(s+1616.65\right)\left({\left(s+840.76\right)}^{2}+56857.98^{2}\right)}{\left(s+2011.00\right)\left(s+2107171.60\right)\left({\left(s+9390.14\right)}^{2}+42766.67^{2}\right)}=\frac{N_{4}\left(s\right)}{D_{4}\left(s\right)}$
	$\frac{{\hat{i}}_{L_{1}}\left(s\right)}{\hat{d}\left(s\right)}=\frac{\left(s+1788.37\right)\left({\left(s-824.70\right)}^{2}+59014.74^{2}\right)}{D_{4}\left(s\right)}$
	$\frac{{\hat{i}}_{L_{2}}\left(s\right)}{{\hat{v}}_{g}\left(s\right)}=\frac{\left(s+2000\right)\left({\left(s+29.18\right)}^{2}+24897.89^{2}\right)}{D_{4}\left(s\right)}$
	$\frac{{\hat{i}}_{L_{2}}\left(s\right)}{\hat{d}\left(s\right)}=\frac{\left(s+2000.00\right)\left({\left(s-1669.26\right)}^{2}+19146.43^{2}\right)}{D_{4}\left(s\right)}$
	$\frac{{\hat{v}}_{C_{1}}\left(s\right)}{{\hat{v}}_{g}\left(s\right)}=\frac{\left(s-37732.47)(s+46662.86)(s+547162.16\right)}{D_{4}\left(s\right)}$
	$\frac{{\hat{v}}_{C_{2}}\left(s\right)}{{\hat{v}}_{g}\left(s\right)}=\frac{\left({\left(s+29.18\right)}^{2}+24897.89^{2}\right)}{D_{4}\left(s\right)}$
	$\frac{{\hat{v}}_{C_{2}}\left(s\right)}{\hat{d}\left(s\right)}=\frac{\left({\left(s-1669.26\right)}^{2}+19146.43^{2}\right)}{D_{4}\left(s\right)}$
	$\frac{{\hat{v}}_{C_{2}}\left(s\right)}{{\hat{i}}_{L_{1}}\left(s\right)}=\frac{\left({\left(s-1669.26\right)}^{2}+19146.43^{2}\right)}{\left(s+1788.37\right)\left({\left(s-824.70\right)}^{2}+59014.74^{2}\right)}$
	$\frac{{\hat{v}}_{C_{2}}\left(s\right)}{{\hat{i}}_{L_{2}}\left(s\right)}=\frac{\left({\left(s-1669.26\right)}^{2}+19146.43^{2}\right)}{\left(s+2000.00\right)\left({\left(s-1669.26\right)}^{2}+19146.43^{2}\right)}$
	${\hat{Z}}_{in}\left(s\right)=\frac{D_{4}\left(s\right)}{\left(s+2001.98\right)\left(s+2107184.16\right)\left({\left(s+499.48\right)}^{2}+43880.90^{2}\right)}$
	${\hat{Z}}_{out}\left(s\right)=\frac{\left(s+2120818.47\right)\left({\left(s+2572.21\right)}^{2}+30838.87^{2}\right)}{D_{4}\left(s\right)}$
Zeta (M<0, Test-3)	$\frac{{\hat{i}}_{L_{1}}\left(s\right)}{{\hat{v}}_{g}\left(s\right)}=\frac{\left(s+3489.33\right)({\left(s+5746.21\right)}^{2}+122263.61^{2})}{\left(s+3622.05\right)\left(s+2223842.84\right)\left({\left(s+30249.00\right)}^{2}+95764.65^{2}\right)}=\frac{N_{5}\left(s\right)}{D_{5}\left(s\right)}$
	$\frac{{\hat{i}}_{L_{1}}\left(s\right)}{\hat{d}\left(s\right)}=\frac{\left(s+3552.63\right)\left({\left(s-10081.49\right)}^{2}+132036.58^{2}\right)}{D_{5}\left(s\right)}$
	$\frac{{\hat{i}}_{L_{2}}\left(s\right)}{{\hat{v}}_{g}\left(s\right)}=\frac{\left(s+2000\right)\left({\left(s+291.81\right)}^{2}+78733.56^{2}\right)}{D_{5}\left(s\right)}$
	$\frac{{\hat{i}}_{L_{2}}\left(s\right)}{\hat{d}\left(s\right)}=\frac{\left(s+2000.00\right)\left({\left(s-16692.26\right)}^{2}+58438.68^{2}\right)}{D_{5}\left(s\right)}$
	$\frac{{\hat{v}}_{C_{1}}\left(s\right)}{{\hat{v}}_{g}\left(s\right)}=\frac{\left(s-37732.47)(s+46662.86)(s+547162.16\right)}{D_{5}\left(s\right)}$
	$\frac{{\hat{v}}_{C_{2}}\left(s\right)}{{\hat{v}}_{g}\left(s\right)}=\frac{({\left(s+291.81\right)}^{2}+78733.56^{2})}{D_{5}\left(s\right)}$
	$\frac{{\hat{v}}_{C_{2}}\left(s\right)}{\hat{d}\left(s\right)}=\frac{({\left(s-16692.62\right)}^{2}+58438.68^{2})}{D_{5}\left(s\right)}$
	$\frac{{\hat{v}}_{C_{2}}\left(s\right)}{{\hat{i}}_{L_{1}}\left(s\right)}=\frac{({\left(s-16692.62\right)}^{2}+58438.68^{2})}{\left(s+3552.63\right)\left({\left(s-10081.49\right)}^{2}+132036.58^{2}\right)}$
	$\frac{{\hat{v}}_{C_{2}}\left(s\right)}{{\hat{i}}_{L_{2}}\left(s\right)}=\frac{({\left(s-16692.62\right)}^{2}+58438.68^{2})}{\left(s+2000.00\right)\left({\left(s-16692.26\right)}^{2}+58438.68^{2}\right)}$
	${\hat{Z}}_{in}\left(s\right)=\frac{D_{5}\left(s\right)}{\left(s+3640.91\right)\left(s+2102679.05\right)\left({\left(s+1932.57\right)}^{2}+102995.44^{2}\right)}$
	${\hat{Z}}_{out}\left(s\right)=\frac{\left(s+2236967.59\right)\left({\left(s+24497.65\right)}^{2}+92082.31^{2}\right)}{D_{5}\left(s\right)}$

**Table 8:** Small signal results in Ćuk converter without coupled inductors.

$v_{g}$	Model	${\bar{i}}_{L_{1}}$ [A]	${\bar{i}}_{L_{2}}$ [A]	${\bar{v}}_{C_{1}}$ [V]	${\bar{v}}_{C_{2}}$ [V]
	Switched	0.2566	0.1518	24.1798	15.1798
9 V	Theoretical	0.2553	0.1516	24.1587	15.1587
	RE [%]	0.51	0.13	0.09	0.14
	Switched	0.2851	0.1687	26.8665	16.8665
10 V	Theoretical	0.2837	0.1684	26.8430	16.8430
	RE [%]	0.49	0.18	0.09	0.14

**Table 9:** Components description of the reconfigurable power converter.

Component	Description	Type
*S*	Power MOSFET	IRFB4510PBF
$L_{A}$	Inductor	Coilcraft’s Hexa-Path HPH4-0140L, 23.7 μH
$L_{B}$	Inductor	Wurth Elektronik 7443551920, 9.2 μH
$L_{C}$	Inductor	Wurth Elektronik 74435584700, 47 μH
$C_{1}$ (Test 1, 2)	Multilayer Ceramic Capacitor	TDK C5750X7S2A106M230KB, 2 × 10μF
$C_{1}$ (Test 3)	Multilayer Ceramic Capacitor	Murata GRM31C2C1H104JA01L, 5 × 100 nF
$C_{2}$ (Test 1, 2, 3)	Multilayer Ceramic Capacitor	TDK C5750X7S2A106M230KB, 2 × 10μF
$C_{d}$ (Test 2)	Multilayer Ceramic Capacitor	TDK C5750X7S2A106M230KB, 5 × 10μF
$R_{d}$ (Test 2)	Damping Resistor	Panasonic ERX5SJ1R5, 1.5 Ω, 5 W

**Table 10:** Theoretical transfer functions of the Zeta converter presented in [29].

Description	Transfer Function
Theoretical proposed	$\frac{{\hat{v}}_{C_{2}}\left(s\right)}{{\hat{v}}_{g}\left(s\right)}=\frac{{\left(s+4348.24\right)}^{2}+117485.36^{2}}{\left(s+421.48\right)\left(s+544303.36\right)\left({\left(s+1292.38\right)}^{2}+81548.65^{2}\right)}=\frac{N_{1}\left(s\right)}{D_{1}\left(s\right)}$
	$\frac{{\hat{v}}_{C_{2}}\left(s\right)}{\hat{d}\left(s\right)}=\frac{{\left(s+8301.02\right)}^{2}+100829.19^{2}}{D_{1}\left(s\right)}$
Theoretical [29]	$\frac{{\hat{v}}_{C_{2}}\left(s\right)}{{\hat{v}}_{g}\left(s\right)}=\frac{s^{2}+117564.93^{2}}{\left(s+417.24\right)\left(s+339468.02\right)\left({\left(s+1333.55\right)}^{2}+81988.22^{2}\right)}=\frac{N_{2}\left(s\right)}{D_{2}\left(s\right)}$
	$\frac{{\hat{v}}_{C_{2}}\left(s\right)}{\hat{d}\left(s\right)}=\frac{{\left(s+5955.04\right)}^{2}+100994.30^{2}}{D_{2}\left(s\right)}$

## Data Availability

Not applicable.

## Abbreviations

The following abbreviations are used in this manuscript:

CCM	Continuous Conduction Mode
DCM	Discontinuous Conduction Mode
HIL	Hardware In The Loop
PFC	Power Factor Correction
SEPIC	Single-Ended Primary-Inductor Converter
